# Single-Photon Sources Based on Novel Color Centers in Silicon Carbide P–I–N Diodes: Combining Theory and Experiment

**DOI:** 10.1007/s40820-021-00600-y

**Published:** 2021-03-06

**Authors:** Igor A. Khramtsov, Dmitry Yu. Fedyanin

**Affiliations:** Laboratory of Nanooptics and Plasmonics, Center for Photonics and 2D Materials, Moscow Institute of Physics and Technology, 141700, Dolgoprudny, Russian Federation

**Keywords:** Color centers, Electron capture cross section, Single-photon emitting diodes, Single-photon electroluminescence, Charge state control

## Abstract

**Highlights:**

- Theory of electrically driven single-photon sources based on color centers in silicon carbide p–i–n diodes.
- New method of determining the electron and hole capture cross sections by an optically active point defect (color center) from the experimental measurements of the single-photon electroluminescence rate and second-order coherence.
- The developed method is based on the measurements at the single defect level. Therefore, in contrast to other approaches, one point defect is sufficient to measure its electron and hole capture cross sections.

**Abstract:**

Point defects in the crystal lattice of SiC, known as color centers, have recently emerged as one of the most promising single-photon emitters for non-classical light sources. However, the search for the best color center that satisfies all the requirements of practical applications has only just begun. Many color centers in SiC have been recently discovered but not yet identified. Therefore, it is extremely challenging to understand their optoelectronic properties and evaluate their potential for use in practical single-photon sources. Here, we present a theoretical approach that explains the experiments on single-photon electroluminescence (SPEL) of novel color centers in SiC p–i–n diodes and gives the possibility to engineer highly efficient single-photon emitting diodes based on them. Moreover, we develop a novel method of determining the electron and hole capture cross sections by the color center from experimental measurements of the SPEL rate and second-order coherence. Unlike other methods, the developed approach uses the experimental results at the single defect level that can be easily obtained as soon as a single-color center is identified in the i-type region of the SiC p–i–n diode.

**Graphic Abstract:**

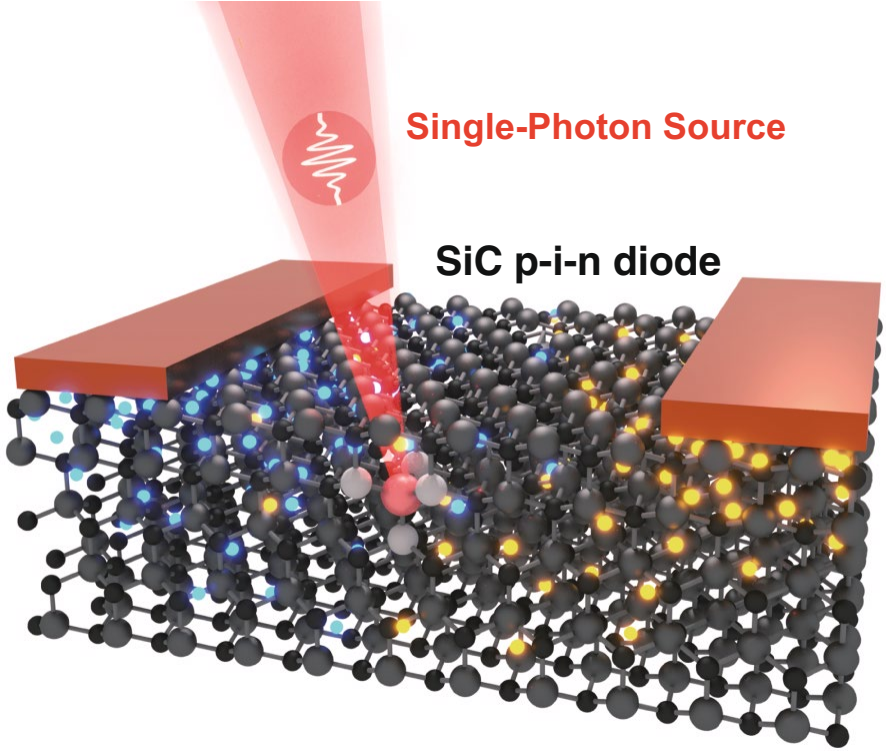

## Introduction

In spite of the significant progress in the development of quantum technologies in the past decade, the lack of bright and efficient true single-photon sources that can operate outside of a research laboratory impedes the implementation of many practical quantum information, metrology, and sensing devices, ranging from unconditionally secure communication lines to optical quantum computers. Although attenuated lasers can partially substitute for true single-photon sources (for example, in quantum cryptography applications [1–3]), they do not allow to use the full potential of quantum technologies since they do not provide single photons [4, 5]. It has been demonstrated that single photons can be produced at a rate of up to 10^6^ photons per second using spontaneous parametric down-conversion [6–8]. However, this approach does not allow to generate single photons on demand, which greatly limits its potential for practical applications. In this regard, single-photon sources based on quantum systems capable of single-photon emission are in highest demand [9]. Ideally, the quantum system should operate under ambient conditions and be excited electrically [9, 10]. However, each quantum system has its own weaknesses. Epitaxial quantum dots demonstrate remarkable emission properties under electrical pumping [11, 12] but operate only at low temperatures [13]. Color centers in diamond, silicon carbide, and related materials can operate at room and higher temperatures under both optical and electrical pumping [14–26]. Most of these point defects in the crystal lattice of wide-bandgap semiconductors are free from blinking and bleaching and have a short radiative lifetime of the excited state. However, their electrical excitation is complicated due to the low density of free carriers in wide-bandgap semiconductors [27–32]. Meanwhile, electrical pumping is the only possibility to achieve high energy efficiency, integrability, and scalability of single-photon sources [10, 33–35]. In this regard, silicon carbide is probably the most promising host material for color centers since the expected brightness of electrically pumped color centers in silicon carbide is significantly higher than in diamond, 2D hexagonal boron nitride, and many other materials [23, 36, 37]. Compared to diamond, silicon carbide is a relatively new material in quantum optics, and, therefore, only a few color centers have been identified and studied in detail [20]. Nevertheless, it has already been found that some yet unidentified point defects emit at telecom wavelengths [38, 39]. These findings make single-photon sources based on silicon carbide even more attractive for practical applications, and further research in this direction is highly demanding.

In this work, using a rigorous theoretical approach and self-consistent numerical simulations, we explain the recent experiments on single-photon electroluminescence of the yet unidentified color centers in 4H-SiC p–i–n diodes. We analyze how the inherent properties of the color center affect the measurements and, moreover, develop a novel method of determining the electron and hole capture cross sections by the color center from the electroluminescence measurements at the single defect level.

## Results and Discussion

### 4H-SiC Single-Photon Emitting Diode

**Fig. 1:**
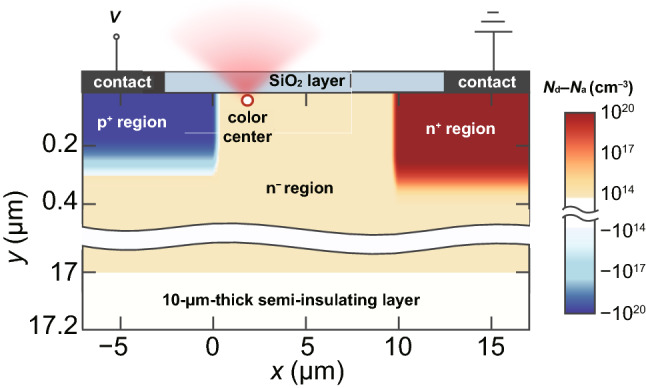
Schematic of the single-photon emitting diode based on a color center in a lateral 4H-SiC p^+^–n^−^–n^+^ diode and spatial distribution of donors and acceptors in the device

Figure 1 shows a schematic of the single-photon emitting diodes (SPEDs) based on a color center in the n^−^-type region of the lateral 4H-SiC p^+^–n^−^–n^+^ diode. The thickness of the n^−^-type layer is equal to 17 μm, which is on top of the 10-μm-thick 4H-SiC semi-insulating layer with almost zero free carrier density. The distance between the p^+^-type and n^+^-type regions is equal to 10 μm, and the thicknesses of these heavily doped regions are about 250 nm and 300 nm, respectively. The spatial distribution of donors and acceptors is shown in Fig. 1. The donor compensation ratio by acceptor-type impurities and defects in the n-type regions and acceptor compensation ratio by donor-type impurities and defects in the p-type region are assumed to be 1% [28]. The thickness of the oxide layer on top of the n^−^-type region is 45 nm. The color center is located in the n^−^-type region near the 4H-SiC/SiO_2_ interface. This SPED configuration was recently experimentally studied in Refs. [24, 25], and several new color centers showing both electroluminescence and photoluminescence have been found, particularly the one that emits at wavelengths 700–850 nm [24]. The origin of these point defects in the crystal lattice of 4H-SiC has not been identified yet, and very little is known about their properties. In this regard, it is important to understand their optoelectronic properties and gain knowledge about the potential of these and other novel color centers in SiC for practical applications in quantum optics and optoelectronics.

Since the origin of the studied color center has not been identified, its charge states and electronic energy level structures of these charges are not known yet. Therefore, we perform here a comprehensive theoretical and numerical analysis of the single-photon electroluminescence (SPEL) of novel color centers in 4H-SiC by extending the theory developed by Fedyanin et al. [23, 40, 41]. We assume that the color center has two charge states: (0) and (− 1),— that are involved in the electroluminescence process. This model has been successfully used to describe the electroluminescence of NV centers and SiV centers in diamond [40, 41]. Moreover, the only known color center that has a positively charged state which is involved in the electroluminescence process is the silicon antisite defect in 4H-SiC [22, 23]. Therefore, our assumption is the most natural in the case of the unidentified center.

**Fig. 2:**
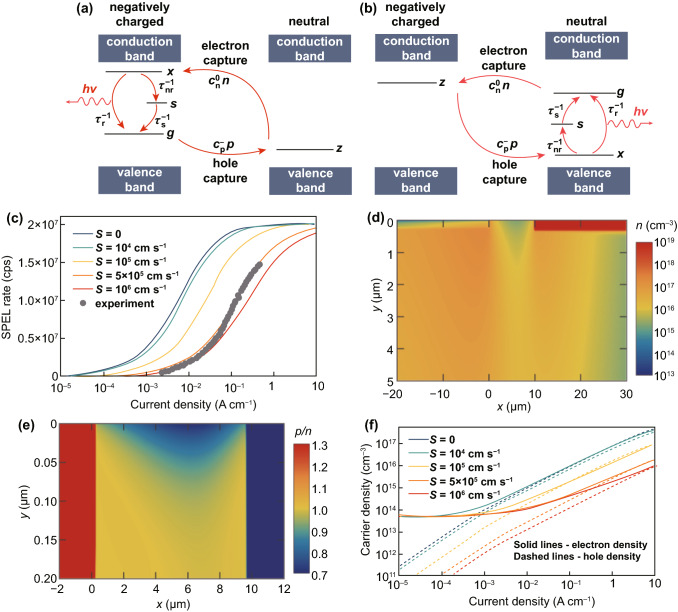
**a** Diagram illustrating the model of the single-photon electroluminescence process for the color center with two charge states (the negatively charged and neutral) that emits from the negatively charged state under electrical pumping. *g*, *x*, and *s* are the populations of the ground, excited, and shelving states of the negatively charged color center; *z* is the population of the neutrally charged state. **b** Diagram illustrating the model of the single-photon electroluminescence process for the color center with a negatively charged state and a neutral charge state that emits from the neutrally charged state under electrical pumping. Since the color center emits after capturing a hole, the energy levels of the neutral color center are plotted for a hole, and, therefore, the excited level is illustrated below the ground level [40, 41]. **c** Simulated SPEL rate versus injection current density per unit diode width for the color center located near the SiO_2_/4H-SiC interface in the n^−^-type region at a distance of 3 μm from the p^+^–n^−^ junction and 10 nm from SiO_2_/4H-SiC interface for different surface recombination velocities *S* at the SiO_2_/4H-SiC interface. Also shown are the experimental points retrieved from Ref. [24] and corrected for the detection efficiency *η*_d_ and collection efficiency *η*_c_ (*η*_d_*η*_c_ = 0.9%). **d** Spatial distribution of free electrons in the n^+^-, n^−^-, and p^+^-type regions of the 4H-SiC p^+^–n^−^–n^+^ diode at a current density per unit device width of *J* = 1.4 A cm^−1^. The surface recombination velocity is equal to 5 × 10^5^ cm s^−1^. **e** Spatial distribution of the ratio of the hole density to electron density at a current density per unit device width of *J* = 1.4 A cm^−1^. The surface recombination velocity is equal to 5 × 10^5^ cm s^−1^. **f** Dependence of the free electron and hole densities in the vicinity of the color center on the injection current for different surface recombination velocities

The SPEL of color centers in SiC is determined by the electron and hole capture processes [23, 40]. The color center can emit a photon either after the electron capture event (like in the case of the SiV center in diamond [40]) or after the hole capture event (like in the case of the NV center in diamond [40, 41]) as shown in Fig. 2a, b. There is no difference in the SPEL rate in the steady state between these two models. The reason for this is that the SPEL process is cyclic, and the SPEL measurements can show only the transition between the excited and ground states of the color center. Therefore, it does not matter whether the color center captures an electron first or captures a hole first between two single-photon emission events. In both models shown in Fig. 2a, b, the SPEL rate is equal to

1 $$R(n,p) = \frac{1}{{1 + \dfrac{{\tau_{{\text{r}}} }}{{\tau_{{{\text{nr}}}} }}}} \times \dfrac{1}{{\dfrac{1}{{c_{{\text{n}}}^{0} n}} + \dfrac{1}{{c_{{\text{p}}}^{ - } p}} + \tau_{{{\text{nr}}}} \dfrac{{\tau_{{{\text{nr}}}} + \tau_{{\text{s}}} }}{{\tau_{{{\text{nr}}}} + \tau_{{\text{r}}} }}}},$$

where *τ*_r_ and *τ*_nr_ are the radiative and non-radiative lifetimes of the excited state and *τ*_s_ is the lifetime of the shelving state, *n* and *p* are the electron and hole densities in 4H-SiC in the vicinity of the color center, and *c*_n_^0^ and *c*_p_^−^ are the electron and hole capture constants. The lifetimes *τ*_r_, *τ*_nr_, and *τ*_s_ have been found using the photoluminescence measurements [24]. The hole capture constant by the negatively charged color center is estimated to be *c*_p_^−^ = 2.8 × 10^−7^ cm^3^s^−1^ using the cascade capture model [23, 40], while the electron capture constant by the color center in the neutral charge state *c*_n_^0^ = 3.6 × 10^−8^ cm^3^ s^−1^ is calculated assuming the electron capture cross section to be about the lattice constant (2 × 10^−15^ cm^2^). A more accurate estimation of the capture rate constants is not possible without knowing the electronic structure of the color center [42, 43].

### Self-Consistent Numerical Simulation of the Single-Photon Emitting Diode

Following the methodology developed in Ref. [23], we self-consistently simulate the SPED shown in Fig. 1 and find the dependence of the SPEL rate on the injection current. The simulation domain is as large as 70 × 27 μm^2^ to take into account the effect produced by long contacts to the n^+^- and p^+^-type regions in the experiment [24]. At the same time, the mesh size near the SiO_2_/4H-SiC interface in the n^−^-type region between the p^+^- and n^+^-type regions is as small as 20 × 5 nm^2^, which ensures accurate simulation of the electron and hole transport in the 4H-SiC SPED. The electron mobilities in the p^+^-, n^−^-, and n^+^-regions are calculated to be 11, 900, and 10 [44–46]. Similarly, the hole mobilities in these regions are calculated to be 25, 140, and 20. The dielectric permittivity is averaged over all directions and equals 10 [47]. Other parameters used in the numerical simulations can be found in Refs. [23, 48].

Figure 2c shows the simulated dependence of the SPEL rate on the injection current for the color center located near the SiO_2_/4H-SiC interface in the n^−^-type region at a distance of 3 μm from the p^+^–n^−^ junction. Since the surface recombination velocity *S* was the only unknown parameter, it was varied in the simulations to better fit the experimental data. As can be seen, the results of the numerical simulations demonstrate almost ideal coincidence with the measurements at *S* = 5 × 10^5^ cm s^−1^. Although the obtained value of the recombination velocity is relatively high, it is consistent with experimental measurements on some 4H-SiC devices [49]. Nevertheless, we expect the surface recombination velocity to be slightly lower. Since the surface recombination velocity is not reported in Ref. [24] and the theory gives only approximate value of the electron and hole capture cross sections, below, we discuss how the surface recombination velocity and free carrier capture cross section affect the single-photon electroluminescence measurements.

### Electron Capture Cross Section

It is important to note that in silicon carbide p–i–n diodes, the current through the device is determined only by the Shockley–Read–Hall recombination in the i-type region and at the 4H-SiC/SiO_2_ interface:

2 $$J = \int {\frac{n(x,y)p(x,y)}{{\tau_{{\text{p}}} (n(x,y) - n_{0} ) + \tau_{{\text{n}}} (p(x,y) - p_{0} )}}{\text{d}}x\,{\text{d}}y,}$$

where *τ*_n_ and *τ*_p_ are the lifetimes of electrons and holes, *n*_0_ and *p*_0_ are the electron and hole densities when the Fermi level coincides with the energy level of the traps. Electrons that flow from the n-type region do not reach the p-type metal contact (Fig. 2d). Instead, in the i-type region, they recombine with holes injected from the p-type region. Since the single act of recombination involves one electron and one hole, the density of electrons and holes in the i-type region of the 4H-SiC p–i–n diode is equal to each other under the forward bias conditions (Fig. 2e). Thus, the densities of both electrons and holes at moderate and high injection levels are unambiguously determined by the injection current. In the 4H-SiC p^+^–n^−^–n^+^ diode shown in Fig. 1, at zero bias voltage, the electron density in the vicinity of the color center in the n^−^-type region is of the order of 10^12^–10^13^ cm^−3^, depending on the position of the color center, which is much larger than the hole density. However, as the bias voltage increases, the hole density rapidly increases and approaches the electron density (Fig. 2f). At current densities per unit device width above 1 A cm^−1^, the electron and hole densities are almost equal to each other.

**Fig. 3:**
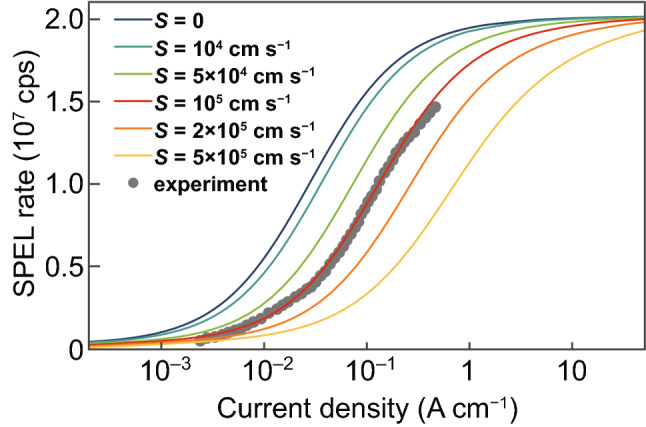
Simulated SPEL rate versus injection current density per unit diode width for the color center located near the SiO_2_/4H-SiC interface in the n^−^-type region at a distance of 3 μm from the p^+^–n^−^ junction and 10 nm from SiO_2_/4H-SiC interface for different surface recombination velocities *S* at the SiO_2_/4H-SiC interface. The electron capture cross section by a neutrally charged color center *c*_n_^0^ is equal to 7.2 × 10^−9^ cm^3^ s^−1^

The fact that *n* = *p* in the i-type region of the 4H-SiC p^+^–n^−^–n^+^ diode at moderate and high injection currents allows to simplify Eq. (1). If the properties of the defects at the SiO_2_/4H-SiC interface are known, i.e., the surface recombination velocity is known, it becomes possible to retrieve (1/*c*_p_^−^ + 1/*c*_n_^0^)^−1^ from the experimental data by fitting the dependence of the SPEL rate on the injection current using numerical simulations (Fig. 3). Even if the properties of the SiO_2_/4H-SiC interface are not well known, the theory combined with the numerical simulations allows to estimate the upper and lower bounds of the carrier capture cross section by the color center. Moreover, since the color center in the negatively charged state attracts positively charged holes, the hole-capture constant *c*_p_^−^ by the negatively charged color center is much larger than the electron capture cross section by the neutral color center. Therefore, (1/*c*_p_^−^ + 1/*c*_n_^0^)^−1^ ≈ *c*_n_^0^, which allows to find the electron capture constant.

If the surface recombination velocity is lower than 10^4^ cm s^−1^, it does not affect the dependence of the SPEL rate on the injection current, as shown in Fig. 2c. In this case, the electron capture cross section is equal to about 2 × 10^−9^ cm^3^ s^−1^, which gives the lower bound on *c*_n_^0^. Using the fact that the surface recombination velocity at the 4H-SiC/SiO_2_ interface should not exceed 10^6^ cm s^−1^ [49–51], we obtain the upper bound on *c*_n_^0^ of 7.2 × 10^−8^ cm^3^ s^−1^. We expect the surface recombination velocity *S* to be of the order of 10^5^ cm s^−1^ [49]. However, since *S* has not been measured for the experimentally studied device, in Table 1, we summarize how the surface recombination velocity used in the numerical simulations affects the electron capture constant *c*_n_^0^ and electron capture cross section *σ*_n_^0^ by the neutral color center.

**Table 1:** Dependence of the electron capture constant *c*_n_^0^ and the electron capture cross section *σ*_n_^0^ by the neutral color center and the hole capture constant *c*_p_^−^ and hole capture cross section *σ*_p_^−^ by the negatively charged color center on the surface recombination velocity *S* used in the numerical simulations

*S* (cm s^−1^)	*c*_n_^0^ (cm^3^ s^−1^)	*σ*_n_^0^ (cm^2^)	*c*_p_^−^ (cm^3^ s^−1^)	*σ*_p_^−^ (cm^2^)
0	1.5 × 10^−9^	8.3 × 10^−17^	2.9 × 10^−8^	2.6 × 10^−15^
10^4^	2.0 × 10^−9^	1.1 × 10^−16^	4.0 × 10^−8^	3.5 × 10^−15^
2 × 10^4^	2.4 × 10^−9^	1.3 × 10^−16^	4.7 × 10^−8^	4.2 × 10^−15^
5 × 10^4^	4.0 × 10^−9^	2.2 × 10^−16^	8.0 × 10^−8^	7.1 × 10^−15^
1 × 10^5^	6.9 × 10^−9^	3.8 × 10^−16^	1.5 × 10^−7^	1.3 × 10^−14^
2 × 10^5^	1.3 × 10^−8^	7.4 × 10^−16^	3.4 × 10^−7^	3.0 × 10^−14^
5 × 10^5^	3.6 × 10^−8^	2.0 × 10^−15^	8.4 × 10^−7^	7.4 × 10^−14^
1 × 10^6^	6.8 × 10^−8^	3.8 × 10^−15^	1.5 × 10^−6^	1.3 × 10^−13^

### Hole Capture Cross section

Besides the dependence of the SPEL rate on the injection current, the *g*^(2)^ function of a single electrically pumped color center is usually measured in the experiment [17, 18, 22, 24]. Despite that in most studies, the *g*^(2)^ function is used just to prove that the source generates single-photon states, it gives a lot of information about the electro-optical properties of the color center [23, 41]. The *g*^(2)^ function of an electrically pumped color center with two charge states shown in Fig. 2a can be found by solving the equations for the population dynamics of the ground and excited states of the neutral and negatively charged color center using the following initial conditions: the population of the negative ground state *g* is equal to 1 and the populations of the negative excited state (*x*), negative shelving state (*s*) and neutral state (*z*) are equal to 0 at *t* = 0 [41]. At an SPEL rate of higher than 10 photons s^−1^, we can neglect thermal excitations, which greatly simplifies the system of equations that describes the population dynamics of the states of the color center [23]:

3 $$\left\{ {\begin{array}{*{20}l} {\dfrac{{{\text{d}}x(t)}}{{{\text{d}}t}} = z(t)c_{{\text{n}}}^{0} n(J) - x(t)\left( {\dfrac{1}{{\tau_{{\text{r}}} }} + \dfrac{1}{{\tau_{{{\text{nr}}}} }}} \right),} \hfill \\ {\dfrac{{{\text{d}}s(t)}}{{{\text{d}}t}} = \dfrac{x(t)}{{\tau_{{{\text{nr}}}} }} - \dfrac{s(t)}{{\tau_{{\text{s}}} }},} \hfill \\ {\dfrac{{{\text{d}}g(t)}}{{{\text{d}}t}} = \dfrac{x(t)}{{\tau_{{\text{r}}} }} + \dfrac{s(t)}{{\tau_{{\text{s}}} }} - g(t)c_{{\text{p}}}^{ - } p(J),} \hfill \\ {\dfrac{{{\text{d}}z(t)}}{{{\text{d}}t}} = g(t)c_{{\text{p}}}^{ - } p(J) - z(t)c_{{\text{n}}}^{0} n(J).} \hfill \\ \end{array} } \right.$$

**Fig. 4:**
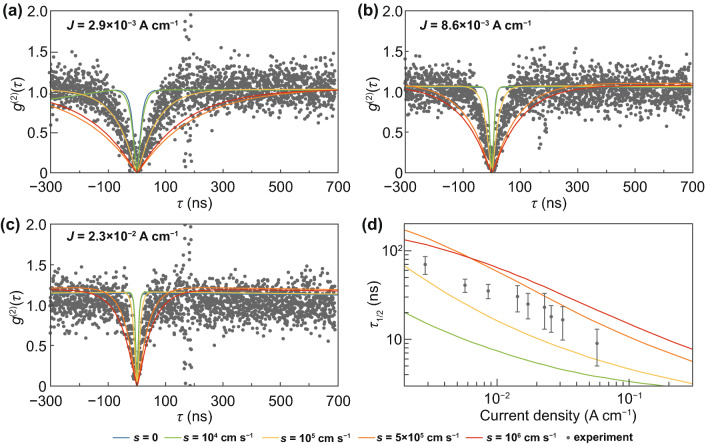
**a–c** Numerically simulated *g*^(2)^ functions and the experimental *g*^(2)^ curves retrieved from Ref. [24] at current densities of 2.9 (**a**), 8.6 (**b**), and 23 (**c**) mA cm^−1^. **d** Comparison between the numerically simulated *g*^(2)^ function half-rise time and the *g*^(2)^ function half-rise time obtained in the experiment. In all panels, the *g*^(2)^ functions are simulated using the hole capture constant given by the cascade capture model *c*_p_^−^ = 2.8 × 10^−7^ cm^3^ s^−1^ and the electron capture constant from Table 1

The solution of this system of equations is a triple-exponential function [23]. It should be noted that we obtain absolutely the same solution in the case of the model shown in Fig. 2b since for the photon emission process, it does not matter whether the color center captures an electron first or a hole first between two single-photon emission events. The analytical solution is, however, difficult and practically useless, since the obtained expressions are very bulk and are difficult to analyze. Therefore, we employ the numerical technique. Figure 4 shows the experimentally measured *g*^(2)^ functions and *g*^(2)^ functions simulated at different surface recombination velocities assuming the hole capture rate constant to be given by the cascade capture model (*c*_p_^−^ = 2.8 × 10^−7^ cm^3^ s^−1^, as discussed above). For every surface recombination velocity, we used the electron capture constant *c*_n_ obtained by fitting the dependence of the SPEL rate on the injection current (see Table 1). It can be seen that the theoretical curves do not give a good fit to the experimental data at a fixed surface recombination velocity (Fig. 4a–c). From the data at low injection currents, it seems that *S* should be about 10^5^ cm/s, while the high current data show that *S* should be higher than 5 × 10^5^ cm s^−1^. To better understand this, we introduce the *g*^(2)^ function half-rise time *τ*_1/2_ and compare the results of the numerical simulations with the experimental data (Fig. 4d). It can be seen that at a hole capture constant of 2.8 × 10^−7^ cm^3^ s^−1^, none of the curves plotted in Fig. 4d gives a good fit to the experimental points.

**Fig. 5:**
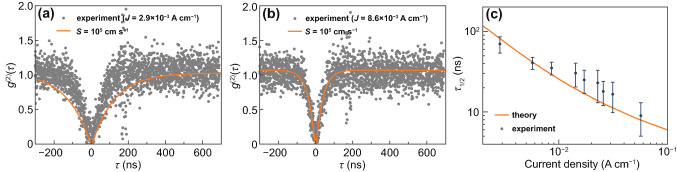
**a, b** Theoretical *g*^(2)^ functions simulated at *c*_p_^−^ = 1.5 × 10^−7^ cm^3^ s^−1^ and experimental *g*^(2)^ curves retrieved from Ref. [24] at current densities of 2.9 (**a**) and 8.6 (**b**) mA cm^−1^. **c** Half-rise time of the experimentally measured *g*^(2)^ function and fit obtained using numerical simulations of the 4H-SiC single-photon emitting diode. In all panels, *S* = 10^5^ cm s^−1^, *c*_p_^−^ = 1.5 × 10^−7^ cm^3^ s^−1^, and *c*_n_^0^ = 6.9 × 10^−9^ cm^3^ s^−1^ for the theoretical curves

Since the electron capture constant has already been found from the dependence of the SPEL rate on the injection current, we can use the theory and numerical simulations to find the hole capture constant from the experimental data for the *g*^(2)^ function. Although the system of Eq. (3) is difficult to analyze, it has been demonstrated that in the absence of the shelving state, the characteristic time of the *g*^(2)^ function is equal to 1/(*c*_n_^0^*n* + *c*_p_^−^*p*) at moderate and low injection levels [41]. Since *c*_n_^0^ ≪ *c*_p_^−^, the *g*^(2)^ function is mostly determined by the hole capture process rather than the electron capture process. Hence, we can find the hole capture rate constant *c*_p_^−^ from the experimentally measured *g*^(2)^ functions. Since at high surface recombination velocities (> 10^5^ cm s^−1^), the hole density is slightly lower than the electron density in the n^−^-type region near the 4H-SiC/SiO_2_ interface (Fig. 2e, f), we take into account the non-zero *c*_n_^0^ in our direct numerical simulations and fit the *g*^(2)^ functions. The obtained hole capture constants at different surface recombination velocities are summarized in Table 1. Figure 5 clearly shows that the new values of the hole capture constant give much better coincidence with the experimental results than the hole capture constant calculated using the cascade capture model, at both low and high injection currents.

### Charge States of the Color Center

**Fig. 6:**
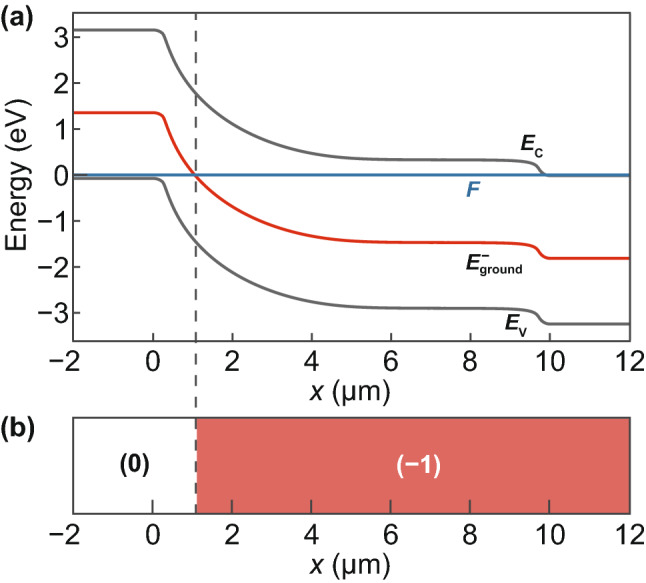
**a** Energy-band diagram of the lateral 4H-SiC p^+^–n^−^–n^+^ diode shown in Fig. 1 along the line *y* = 10 nm in equilibrium (*V* = 0), *F* is the Fermi level. The (0|− 1) charge state transition level is illustrated to be 1.9 eV below the conduction band edge of 4H-SiC. **b** Dependence of the charge of the color center on its position in the 4H-SiC p^+^–n^−^–n^+^ diode at *y* = 10 nm for the (0|− 1) charge state transition level shown in panel **a**

We should note that although the developed theoretical approach allows to explain the experimental results, reproduce the dependence of the SPEL rate on the injection current and the *g*^(2)^ functions at different currents, and even find the electron and hole capture cross sections by the color center, it does not give the possibility to find from which charge state the color center emits photons under electrical pumping since the SPEL rate and the *g*^(2)^ functions are the characteristics of the photon emission process, which are not sensitive to the order of the hole capture and electron capture events between two photon emission events as discussed above. Therefore, to find from which charge state the color center emits, additional measurements are required. Figure 6a shows the energy-band diagram of the 4H-SiC SPED in equilibrium. The charge state of the color center at position (*x*, *y*) is determined by the position of the Fermi level at point (*x*, *y*) with respect to the energy level of the ground state of the color center (Fig. 6a, b) [52–56]. The occupation of the (− 1) charge state in a single-electron approximation is given by [52]:

4 $$f_{ - 1} (F) = \dfrac{1}{{1 + \dfrac{{g_{0} }}{{g_{1} }}\exp \left( {\dfrac{{E_{{{\text{ground}}}}^{ - } - F}}{kT}} \right)}},$$

where *g*_0_ and *g*_1_ are the degeneracy factors of the (0) and (− 1) charge states, respectively, *F* is the Fermi level in 4H-SiC in the vicinity of the color center, *E*^−^_ground_ is the ground level of the color center in the (− 1) charge state, and *kT* is the thermal energy. If *F* is below *E*^−^_ground_, the color center in the (0) charge state, and vice versa, if *F* is above *E*^−^_ground_, the color center is in the (− 1) charge state. In this regard, *E*^−^_ground_ is typically referred to as the (0|− 1) charge state transition level. If the color center emits from the (− 1) charge state (Fig. 2a), the (0|− 1) charge state transition level should lie more than 1.9 eV below the conduction band edge, since spectral measurements of the studied color center show that energy of the emitted photon can be as high as 1.77 eV [24], the excited state should lie below the conduction band edge to be able to capture an electron from the conduction band, and we do not observe any sign of thermal emission of electrons from the excited state to the conduction band of silicon carbide in the numerical simulations that reproduce the experimental results. Therefore, in equilibrium, the color center should show photoluminescence only if it is to the right of *x* = 1 μm (Fig. 6).

Similarly, if the color center emits from the (0) charge state (Fig. 2b), the (0|− 1) charge state transition level (the ground level of the (− 1) charge state in Fig. 2b, which either coincides with the ground level of the (0) charge state or lies slightly above it) should be more than 1.9 eV above the valence band edge, i.e., less than 1.3 eV below the conduction band edge of silicon carbide. In this case, the color center shows bright photoluminescence in equilibrium if it is on the left of *x* = 1.6 μm. However, if the (0|− 1) charge state transition level is less than 0.5 eV below the conduction band edge, the color center should show bright photoluminescence if it is on the left of *x* = 3.2 μm. The studied color center shows photoluminescence at a distance of about 3 μm from the p^+^–n^−^ junction. Therefore, the choice between the models shown in Fig. 2a, b depends on the ionization energy of the color center. Additional studies of the photoluminescence properties of the considered color center under negative bias voltages [52–56] or studies of color centers of the same type located in the p^+^-type region, n^+^-type region, and in the n^−^-region near the n^−^–n^+^ junction are required to find from which charge state the color center emits. Nevertheless, we should note that it is very unlikely that in 4H-SiC, which has a bandgap energy of 3.23 eV, the (0|− 1) charge state transition level is as close to the conduction band edge as 0.5 eV. Therefore, we should expect the color center to emit from the (− 1) charge state rather than from the (0) charge state under electrical pumping.

Since the charge states of the studied color center are not known, we have also considered the SPEL process that involves the (0) and (+ 1) charge states of the color center, which is similar to the SPEL process of the silicon antisite defect in silicon carbide [23], and, using the developed approach, obtained the electron capture cross section by the positively charged color center and the hole capture cross section by the neutral color center from the experimental measurements of the SPEL rate and *g*^(2)^ functions (Table 2). The theoretical model does not give a good fit to the experimental results for surface recombination velocities higher than 3 × 10^5^ cm s^−1^. At the same time, at lower recombination velocities, the electron capture constant by the positively charged color center in the (0) ↔ (+ 1) model is almost the same as the hole capture constant by the negatively charged color center in the (− 1) ↔ (0) model. Similarly, the capture constants by the neutral color center are almost the same in both models. This is because the electron and hole densities in the vicinity of the color center are almost equal to each other at moderate and high injection levels in the studied 4H-SiC p^+^–n^−^–n^+^ single-photon emitting diode (Fig. 2f). In a 4H-SiC p–i–n single-photon emitting diode, where there are no free carriers near the color center at zero bias, the coincidence between the capture constants by the charged color center in two models would be even better, especially at low surface recombination velocities. Finally, we have considered all possible three-charge-state models of the SPEL process, and the obtained capture cross sections are almost equal to that provided in Tables 1 and 2.

**Table 2:** Electron capture constant *c*_n_^+^ and electron capture cross section *σ*_n_^+^ by the positively charged color center and hole capture constant *c*_p_^0^ and hole capture cross section *σ*_p_^0^ by the neutral color center obtained from the experimental data using the model of the SPEL process that involves the (0) and (+ 1) charges states

*S* (cm s^−1^)	*c*_n_^+^ (cm^3^ s^−1^)	*σ*_n_^+^ (cm^2^)	*c*_p_^0^ (cm^3^ s^−1^)	*σ*_p_^0^ (cm^2^)
0	2.7 × 10^−8^	1.5 × 10^−15^	1.7 × 10^−9^	1.5 × 10^−16^
10^4^	3.5 × 10^−8^	1.9 × 10^−15^	2.1 × 10^−9^	1.9 × 10^−16^
2 × 10^4^	4.1 × 10^−8^	2.3 × 10^−15^	2.6 × 10^−9^	2.3 × 10^−16^
5 × 10^4^	5.8 × 10^−8^	3.2 × 10^−15^	4.3 × 10^−9^	3.8 × 10^−16^
1 × 10^5^	8.4 × 10^−8^	4.7 × 10^−15^	8.5 × 10^−9^	7.5 × 10^−16^
2 × 10^5^	1.2 × 10^−7^	6.9 × 10^−15^	1.7 × 10^−8^	1.5 × 10^−15^

Thus, we can conclude that the developed method gives the possibility to accurately determine the free carrier capture constants by the charged color center and neutral color center, while for determining which charge states are involved in the SPEL process, additional measurements are required.

## Conclusions

In this work, we have presented a theoretical description of the single-photon electroluminescence process of novel color centers in silicon carbide p–i–n diodes, which are considered the most promising electrically driven single-photon sources. We demonstrate that the developed theoretical model allows to explain the experimentally measured dependences. The performed self-consistent numerical simulations reproduce the experimental results on the SPEL rate and the second-order coherence. We show that the electroluminescence properties of the color center, both the SPEL rate and the second-order coherence function, are mostly determined by the free electron and hole capture process by the color center. The proposed theoretical approach for the ab initio estimation of the electron and hole capture rate by the color center is in good agreement with the experimental results. However, there is a small discrepancy between the theory and experiment, which arises due to the difference between the theoretically estimated and actual capture cross sections by the color center. This discrepancy can be used to find the capture cross section from the experimental data.

We developed a novel approach that gives the possibility to evaluate both the electron and hole capture cross sections by the color center using only the measurements of the SPEL rate and the second-order coherence of the electrically pumped color center. Unlike the common techniques, such as the deep-level transient spectroscopy (DLTS), the developed approach allows to retrieve the electron and hole capture cross sections by the color center from the measurements at the single defect level using the numerical simulations. This approach is particularly important in the case of silicon carbide, where it is not possible to create a very high density of defects of only one type. Our approach gives the possibility to study novel color centers in silicon carbide p–i–n diodes as soon as a single defect is isolated and measured. The accuracy of the developed theoretical approach is determined by the accuracy in determining the electrical properties of silicon carbide p–i–n diode, particularly the surface recombination velocity. The latter can significantly affect the measurements. The accuracy of the method with respect to the accuracy of the measured position of the color center, which is typically about ± 0.5 μm, is about 30% at high surface recombination velocities and is less than 20% at surface recombination velocities < 10^4^ cm/s.

The electron and hole capture cross sections obtained using our approach can help to identify the color center, which can be done by comparing the capture cross sections obtained using the DFT and similar techniques with the capture cross sections retrieved from the experiment using our method. The ability to find the capture cross sections by a novel color center is also of crucial importance for the design and development of quantum optoelectronic devices based on novel color centers where charge state switching, control, or stabilization is required, such as for protecting quantum memories based on color centers [56], electrical readout of the spin state of the color center at a high repetition rate [57], pulsed electrical single-photon sources [58], or photocurrent detected magnetic resonance (PDMR) devices [59, 60].
